# An easy-to-use model for screening type 2 myocardial infarction in geriatric patients with acute coronary syndrome: a retrospective cohort study

**DOI:** 10.3389/fmed.2026.1907612

**Published:** 2026-08-27

**Authors:** Yun-yue Luo, Hui Wang, Xiao-ya Ma, Yue Li, Wu-lin Li, Yue Liu, Yu-xin Wang, Wen-hui Kang, Jian-jun Xia, Fei Wang

**Affiliations:** 1 Shanghai University of Medicine & Health Sciences, Shanghai University of Traditional Chinese Medicine, Shanghai, China; 2 Department of Emergency and Critical Care Medicine, Jiading District Central Hospital Affiliated Shanghai University of Medicine & Health Sciences, Shanghai, China; 3 Chest Pain Center, Jiading District Central Hospital Affiliated Shanghai University of Medicine & Health Sciences, Shanghai, China; 4 Department of Quality Management, Sir Run Run Shaw Hospital, Zhejiang University School of Medicine, Zhejiang, China

**Keywords:** acute coronary syndrome, nomogram, prediction model, risk factors, type 2 myocardial infarction

## Abstract

**Objective:**

In this study, we aimed to develop and validate an easy-to-use model for screening type 2 myocardial infarction (T2MI) in geriatric patients with acute coronary syndrome (ACS).

**Method:**

From January 2017 to December 2022, 891 geriatric patients who were clinically diagnosed with ACS and who underwent coronary angiography visited the Chest Pain Center of the Emergency Department at Jiading District Central Hospital affiliated Shanghai University of Medicine & Health Sciences were included. Univariate and multivariate logistic regression analyses were then performed to identify risk factors for T2MI among ACS patients. Subsequently, these factors were used to establish a scoring system, from which a nomogram was developed using R software.

**Result:**

This study included 891 geriatric patients diagnosed with ACS who underwent coronary angiography and met the criteria. Among these patients, 21.3% (190/891) were diagnosed with T2MI. Univariate analysis identified 10 factors significantly associated with T2MI occurrence in geriatric ACS patients (*P* < 0.05). Multivariate logistic regression analysis revealed atrial fibrillation, COPD, anemia, chronic heart failure, presence of atypical symptoms, and non-ST-segment elevation as independent risk factors for T2MI in geriatric ACS patients. The nomogram model, constructed using troponin and the six identified risk factors, demonstrated a AUC of training set of 0.803 [95% confidence interval (CI): 0.765–0.841], indicating good discrimination. Moreover, calibration and clinical decision curve analyses further confirmed the reliability and clinical value of the proposed model.

**Conclusion:**

The predictive nomogram model, based on clinical features of geriatric ACS patients in the emergency department, enables emergency physicians to quickly screen T2MI. Consequently, early identification guides personalized treatment.

## 1 Introduction

Acute myocardial infarction (AMI) is one of the cardiovascular events with the highest mortality and disability rates worldwide. Due to population aging, its incidence has been continuously increasing, making it a significant challenge for global public health (1). The detection of T2MI among AMI patients has increased annually. Some studies have reported that T2MI accounts for 20%–30% of all AMI cases, especially among the geriatric patients with comorbidities (2, 3). Compared with type 1 myocardial infarction (T1MI), patients with T2MI experience higher rates of adverse events and all-cause mortality (4). This significantly reduces the long-term survival quality of geriatric AMI patients.

Although clinical understanding of T2MI has gradually deepened, its identification still faces many challenges (5). The traditional diagnostic process for AMI mainly relies on myocardial injury biomarkers, symptoms, electrocardiography, and coronary angiography; however, these methods have limitations in distinguishing T2MI, particularly in geriatric patients, and clinical diagnosis still depends largely on clinical subjective judgment (6, 7). The emergency department represents a critical setting for the early differentiation of T2MI and the identification of its underlying triggers. Delayed diagnosis and management may result in progressive ischemic myocardial necrosis and increase the risks of heart failure, malignant arrhythmias, cardiogenic shock, and death. Tachyarrhythmia, hypoxemia, and anemia are common triggers of T2MI (4, 8–10), underlying coronary artery disease (CAD) and left ventricular dysfunction may further lower the myocardial ischemic threshold (4, 8, 10–12). Although the combination of clinical symptoms, electrocardiogram (ECG), high-sensitivity cardiac troponin (hs-cTn), and structured decision pathways is recommended by current guidelines for the rapid identification or exclusion of AMI, early screening tools for T2MI remain lacking (5, 13, 14). Elderly patients frequently present with comorbidities including chronic heart failure, anemia, atrial fibrillation (AF), chronic obstructive pulmonary disease (COPD), and coronary artery disease (CAD) (15). The risk of missed diagnosis and misdiagnosis of T2MI is further elevated by their reduced cardiopulmonary reserve, atypical symptoms, and elevated baseline cardiac troponin levels (8–10). Furthermore, the basal concentration, positive predictive value (PPV), and triage efficiency of high-sensitivity cardiac troponin (hs-cTn) may also be influenced by age and renal impairment (13, 14).

Although the Extended Neumann Score may assist in differentiating T1MI from T2MI, its discriminatory capacity is limited, and it was not developed specifically for elderly patients with ACS (16). The T2-risk score is primarily used for the long-term prognostic assessment of patients with confirmed T2MI and is not applicable for early screening at the initial emergency department presentation (17). The HEART, EDACS, and GRACE scores primarily predict the risk of short-term adverse cardiovascular events or mortality; they cannot directly differentiate T1MI, T2MI, and non-ischemic myocardial injury, and their efficiency in low-risk triage among elderly patients is limited (11, 13, 14). Although the CoDE-ACS, MI³, and other machine learning models have improved the capacity for individualized diagnosis and myocardial injury classification, they are generally characterized by a relatively large number of variables, computational complexity, and reliance on serial cardiac troponin measurements or specific assay platforms; furthermore, adequate external validation is still lacking for some of these models (18–20). Although the combination of multiple biomarkers also demonstrates certain discriminatory value, its widespread adoption in rapid emergency department screening is limited by high analytical requirements (21). Therefore, existing tools have not yet adequately met the clinical need for early etiological screening for T2MI in elderly patients presenting to the emergency department with chest pain and suspected ACS. Consequently, early and rapid screening for T2MI in this population is imperative.

The present study was designed to transform the aforementioned multidimensional information into a visualized risk score by means of a nomogram, thereby assisting emergency physicians in utilizing rapidly obtainable clinical variables to screen for the likelihood of T2MI and consequently reducing the risk of missed diagnosis and misdiagnosis.

## 2 Methods

### 2.1 Study population

**Figure 1:**
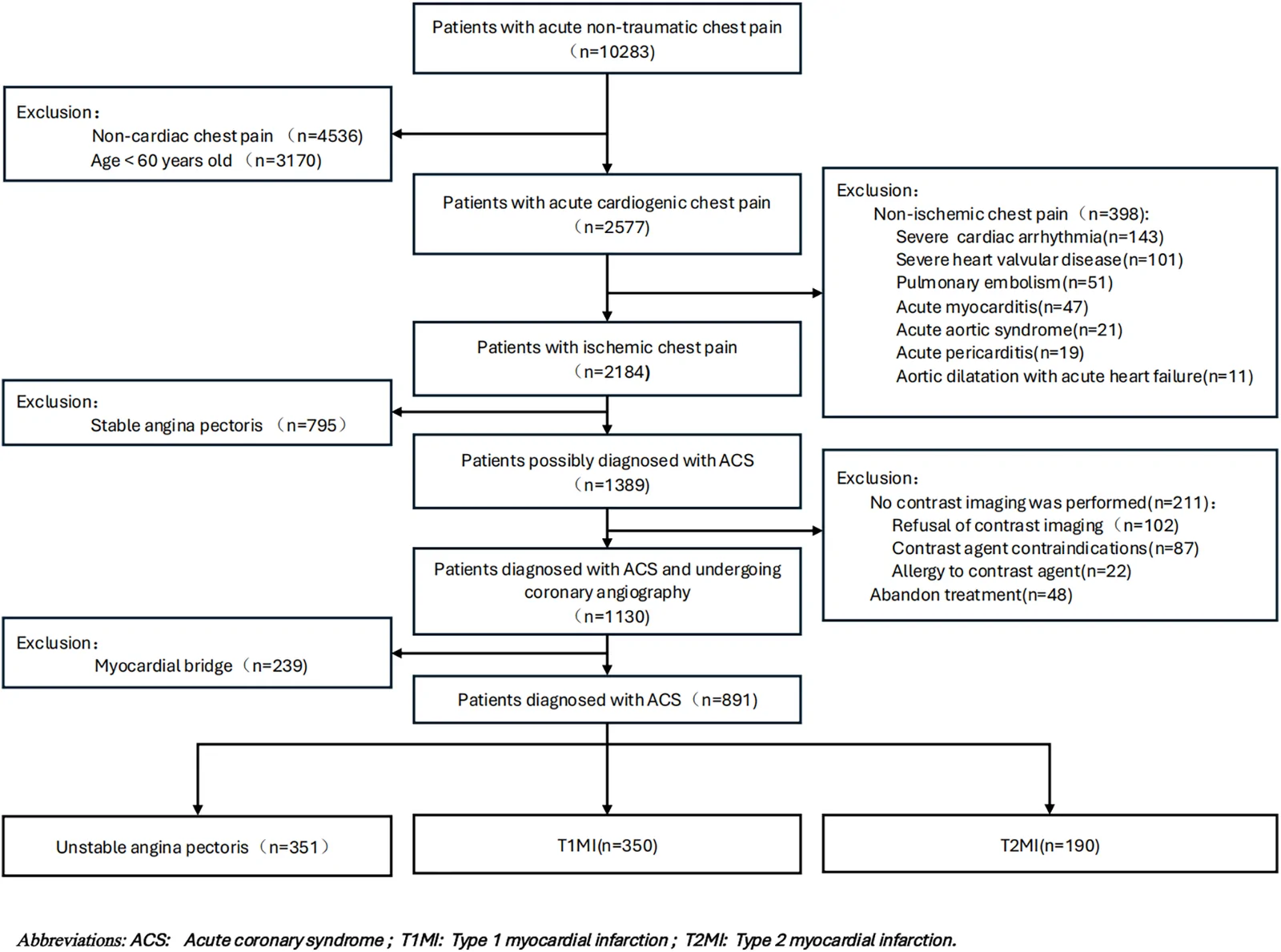
Flowchart for screening patients with chest pain.

A total of 10,283 patients with acute non-traumatic chest pain were consecutively enrolled. These patients visited the Chest Pain Center of the Emergency Department of Jiading District Central Hospital affiliated Shanghai University of Medicine & Health Sciences from January 2017 to December 2022, were consecutively enrolled. All patients underwent clinical assessment. Ultimately, 891 geriatric patients who were clinically diagnosed with ACS and who underwent coronary angiography were included. The flowchart of the study selection process is shown in Figure 1.

#### 2.1.1 Inclusion criteria

1. Patients with non-traumatic chest pain;
2. Age ≥ 60 years old.

#### 2.1.2 Exclusion criteria

1. Age < 60 years old;
2. Non-cardiac chest pain;
3. Non-ischemic cardiac chest pain, including acute pericarditis, acute myocarditis, acute aortic syndrome, pulmonary embolism, dilated cardiomyopathy with acute heart failure, severe arrhythmias, and severe cardiac valve disease;
4. Stable angina pectoris;
5. Contraindications to contrast agents, refusal to undergo contrast imaging, or allergy to contrast agents;
6. Patients diagnosed with a myocardial bridge after contrast imaging;
7. Patients who have discontinued treatment.

### 2.2 Diagnostic criteria

#### 2.2.1 Diagnostic criteria for myocardial infarction

Diagnostic criteria for myocardial infarction (22): Diagnosis is based on dynamic changes in cardiac troponin (cTn) values, with at least one value exceeding the upper reference limit (URL) defined as the 99th percentile, combined with at least one of the following clinical criteria of myocardial ischemia: (1) acute myocardial ischemic symptoms; (2) new ischemic changes on electrocardiogram; (3) development of pathological Q waves; (4) imaging evidence of loss of viable myocardium or new regional wall motion abnormalities; (5) findings from coronary angiography or autopsy.

#### 2.2.2 Definition and adjudication of T2MI (23)

1. Met the diagnostic criteria for Fourth Universal Definition of Myocardial Infarction;
2. Had evidence indicating that the event was unrelated to coronary plaque rupture.

### 2.3 Data collection

The patients included in this study were selected from the internal database of the Chest Pain Center of the Emergency Department of Jiading District Central Hospital affiliated Shanghai University of Medicine & Health Sciences. General information was collected, including demographic data (gender and age), lifestyle factors (smoking and alcohol consumption), and comorbidities such as hypertension; diabetes; hyperlipidemia; chronic heart failure; previous myocardial infarction; chronic obstructive pulmonary disease; Malignant Tumor; Anemia; Chronic Kidney Disease and atrial fibrillation. Additionally, data on emergency symptoms (such as no typical symptoms and Syncope), troponin levels, and ECG.

#### 2.3.1 Definitions of clinical variables

- Hyperlipidemia: In line with commonly applied diagnostic criteria in China, dyslipidemia in adults is diagnosed if any of the following laboratory thresholds are met: total cholesterol (TC) ≥ 6.2 mmol/L, low-density lipoprotein cholesterol (LDL-C) ≥ 4.1 mmol/L, triglycerides (TG) ≥ 2.3 mmol/L, or high-density lipoprotein cholesterol (HDL-C) < 1.0 mmol/L. The presence of any one of these abnormalities is sufficient for a diagnosis. When elevated cholesterol is the predominant abnormality, the condition is termed hypercholesterolemia; when elevated triglycerides predominate, it is termed hypertriglyceridemia (24).
- Chronic heart failure: The diagnosis was made based on current clinical guidelines, requiring the presence of typical symptoms and/or signs of heart failure, along with corroborating evidence from either echocardiography (demonstrating relevant structural or functional cardiac abnormalities) or elevated natriuretic peptide levels (25).
- Anemia: Anemia was defined as a hemoglobin level below the World Health Organization diagnostic thresholds: <130 g/L for men and <120 g/L for non-pregnant women (26).
- Chronic kidney disease: Per the 2024 KDIGO guidelines, chronic kidney disease (CKD) was defined as the presence of kidney structure or function abnormalities that persist for more than three months and impact health. The diagnosis was determined by either a previously documented clinical diagnosis or an estimated glomerular filtration rate (eGFR) of <60 mL/min/1.73 m^2^ (27).
- Atrial fibrillation: Atrial fibrillation (AF) was defined as AF confirmed by electrocardiogram, which included either AF documented on a standard 12-lead electrocardiogram or AF documented on a single-lead or continuous electrocardiographic recording lasting >30 s, with physician verification (28).

### 2.4 Statistical methods

Subsequently, after applying the predefined inclusion and exclusion criteria, 891 geriatric patients diagnosed with ACS were ultimately enrolled. The patients were randomly divided into a training set (*n* = 624) and a validation set (*n* = 267) in a 7:3 ratio. Subsequent analyses were conducted on these datasets.

#### 2.4.1 Data preprocessing

All data preprocessing steps (outlier filtering, missing value imputation and one-hot encoding) were conducted in R 4.3.1. Outliers were processed jointly by clinical judgment and statistical distribution. Records with clinically impossible negative metrics were removed. Statistically, observations exceeding ± 3 standard deviations from the mean were flagged as missing and imputed subsequently to balance clinical reality and data integrity. For missing data, variables missing over 20% were discarded. Mean imputation was used for features with missingness <5%, while Markov Chain Monte Carlo-based multiple imputation was adopted for variables with 5%–20% missing data. No missing values existed in outcome variables (29). All categorical predictors were converted via one-hot encoding.

#### 2.4.2 Descriptive and univariate analyses

For categorical variables, categorical distributions were characterized by counts and corresponding percentages. Intergroup comparisons were primarily implemented via the Pearson *χ*^2^-test. Fisher's exact test was substituted when the prerequisites of the *χ*^2^-test could not be satisfied. Continuous variables conforming to a normal distribution were summarized as mean ± standard deviation, with intergroup disparities analyzed by independent sample *t*-test. Continuous variables with non-normal distributions were described using median together with the 25th and 75th percentiles (interquartile range). The Wilcoxon rank-sum test was utilized for pairwise comparisons of non-normal continuous data.

#### 2.4.3 Variable screening and model construction

Univariate binary logistic regression was conducted on the training set to screen candidate predictors of T2MI with *P* < 0.05. Troponin (cTn), an essential myocardial injury biomarker, was forced into the multivariate model irrespective of its univariate *P*-value to avoid discarding clinically vital indicators by statistical thresholds. All screened candidates were incorporated into multivariate binary logistic regression, where backward stepwise selection retaining predictors at *P* < 0.05 was applied to build a concise, interpretable nomogram. This two-stage approach reduced variable quantity to alleviate overfitting under limited sample size, and comprehensive subsequent validation further guaranteed stable predictive performance.

#### 2.4.4 Model performance evaluation

Model performance evaluation was conducted in both the training set and the internal validation set, using discrimination and calibration as evaluation metric (30). Discrimination metrics included the area under the receiver operating characteristic curve (AUC), sensitivity (true positive rate, TP) and specificity (true negative rate, TN). Model calibration was assessed via calibration curves, Brier score, calibration-in-the-large (CITL) intercept and calibration slope, with 95% CIs of CITL and calibration slope calculated by logistic regression asymptotic standard errors. Decision curves were plotted to evaluate clinical net benefit.

#### 2.4.5 Model visualization

A nomogram was generated to visualize the established logistic regression model. In binary logistic regression, the linear predictor formed by covariates multiplied by their respective regression coefficients determines the predicted event probability. A higher linear predictor value corresponds to a greater probability of T2MI occurrence. This linear combination is defined as the risk score, which can be stratified into distinct risk subgroups by percentile cut-offs to facilitate individualized clinical risk stratification and targeted intervention. The nomogram intuitively quantifies and displays this risk score. All nomogram plotting was implemented via the rms package in R software (version 4.3.1).

All statistical analysis were conducted utilizing R software (Version 4.3.1). A two-tailed significance threshold of *α* = 0.05 was established for all statistical hypothesis tests.

## 3 Result

### 3.1 Baseline characteristics of patients in training and validation set

Among geriatric patients with ACS at the chest pain center of emergency department, 21.3% (190 out of 891) were diagnosed T2MI. After randomly splitting the dataset in a 7:3 ratio, we found that the baseline characteristics of the training and validation sets were similar, as shown in Table 1.

**Table 1:** Baseline characteristics of patients in validation and training set.

Variables	Total (*n* = 891)	validation (*n* = 267)	Training (*n* = 624)	*P*
Demographic data				
Age, Mean ± SD	74.27 ± 8.52	74.54 ± 8.75	74.15 ± 8.43	0.527
Female, *n* (%)	286 (32.10)	88 (32.96)	198 (31.73)	0.719
Current smokers, *n* (%)	56 (6.29)	12 (4.49)	44 (7.05)	0.150
Current drinkers, *n* (%)	14 (1.57)	1 (0.37)	13 (2.08)	0.113
Comorbidities				
Hypertension, *n* (%)	676 (75.87)	206 (77.15)	470 (75.32)	0.558
Diabetes, *n* (%)	288 (32.32)	95 (35.58)	193 (30.93)	0.174
Atrial fibrillation, *n* (%)	112 (12.57)	23 (8.61)	89 (14.26)	0.020
COPD, *n* (%)	75 (8.42)	19 (7.12)	56 (8.97)	0.360
CKD, *n* (%)	90 (10.10)	26 (9.74)	64 (10.26)	0.814
Malignant Tumor, *n* (%)	18 (2.02)	7 (2.62)	11 (1.76)	0.404
Anemia, *n* (%)	39 (4.38)	11 (4.12)	28 (4.49)	0.806
Hyperlipidemia, *n* (%)	27 (3.03)	7 (2.62)	20 (3.21)	0.642
Chronic heart failure, *n* (%)	62 (6.96)	17 (6.37)	45 (7.21)	0.650
Previous myocardial infarction, *n* (%)	25 (2.81)	7 (2.62)	18 (2.88)	0.828
Emergency symptoms				
Syncope, *n* (%)	37 (4.15)	10 (3.75)	27 (4.33)	0.690
No Typical symptoms, *n* (%)	491 (55.11)	141 (52.81)	350 (56.09)	0.367
cTn (ng/mL), M (Q_1_, Q_3_)	0.08 (0.02, 0.70)	0.12 (0.03, 0.93)	0.08 (0.02, 0.61)	0.094
UA, *n* (%)	351 (39.39)	102 (38.20)	249 (39.90)	0.634
Non-ST-segment elevation on ECG, *n* (%)	273 (30.64)	88 (32.96)	185 (29.65)	0.326
MI subtype among patients with AMI				
T1MI, *n* (%)	350 (39.28)	101 (37.83)	249 (39.90)	0.561
T2MI, *n* (%)	190 (21.32)	64 (23.97)	126 (20.19)	0.207

cTn, cardiac Troponin; COPD, Chronic obstructive pulmonary disease; CKD, Chronic Kidney Disease; UA, unstable angina.

### 3.2 Training Set features

In the training set, univariate analysis revealed that T2MI patients were older than non-T2MI patients. They also had higher proportions of females, current smokers, and drinkers. Additionally, more T2MI patients had atrial fibrillation, COPD, chronic kidney disease, anemia, hyperlipidemia, chronic heart failure, old myocardial infarction, and atypical symptoms. Conversely, fewer T2MI patients had hypertension, diabetes, or malignant tumors. Regarding clinical manifestations, patients mainly exhibited atypical chest pain symptoms (31); moreover, the proportion of patients with NSTEMI and non-ST-segment elevation on ECG findings on electrocardiogram was higher, as shown in Table 2.

**Table 2:** Demographic and baseline characteristics of the training set.

Variables	Total (*n* = 624)	non-T2MI (*n* = 498)	T2MI (*n* = 126)	*P*
Demographic data				
Age, Mean ± SD	74.15 ± 8.43	73.62 ± 8.50	76.25 ± 7.81	0.001
Female, *n* (%)	198 (31.73)	150 (30.12)	48 (38.10)	0.086
Current smokers, *n* (%)	44 (7.05)	35 (7.03)	9 (7.14)	0.964
Current drinkers, *n* (%)	13 (2.08)	10 (2.01)	3 (2.38)	1.000
Comorbidities				
Hypertension, *n* (%)	470 (75.32)	376 (75.50)	94 (74.60)	0.834
Diabetes, *n* (%)	193 (30.93)	155 (31.12)	38 (30.16)	0.834
Atrial fibrillation, *n* (%)	89 (14.26)	55 (11.04)	34 (26.98)	<0.001
COPD, *n* (%)	56 (8.97)	35 (7.03)	21 (16.67)	<0.001
CKD, *n* (%)	64 (10.256)	45 (9.036)	19 (15.079)	0.046
Malignant Tumor, *n* (%)	11 (1.76)	9 (1.81)	2 (1.59)	1.000
Anemia, *n* (%)	28 (4.49)	16 (3.21)	12 (9.52)	0.002
Hyperlipidemia, *n* (%)	20 (3.21)	12 (2.41)	8 (6.35)	0.050
Chronic heart failure, *n* (%)	45 (7.21)	26 (5.22)	19 (15.08)	<0.001
Previous myocardial infarction, *n* (%)	18 (2.88)	14 (2.81)	4 (3.17)	1.000
Emergency symptoms				
Syncope, *n* (%)	27 (4.33)	17 (3.41)	10 (7.94)	0.026
No Typical symptoms, *n* (%)	350 (56.09)	265 (53.21)	85 (67.46)	0.004
Non-ST-segment elevation on ECG, *n* (%)	185 (29.65)	101 (20.28)	84 (66.67)	<0.001
CTn (ng/mL), M (Q₁,Q₃)	0.08 (0.02, 0.61)	0.05 (0.02, 0.49)	0.27 (0.06, 0.87)	<0.001
AMI, *n* (%)	375 (60.10)	249 (39.90)	126 (100.00)	<0.001
STEMI	226 (60.27)	171 (68.67)	55 (43.65)	
NSTEMI	149 (39.73)	78 (31.33)	71 (56.35)	

cTn, cardiac Troponin; COPD, Chronic obstructive pulmonary disease; CKD, Chronic Kidney Disease; NSTEMI, Non-ST-Segment Elevation Myocardial Infarction; STEMI, ST-Segment Elevation Myocardial Infarction.

### 3.3 Screening of independent risk factors for T2MI in the training set

In the training set, univariate logistic regression analysis identified several risk factors for T2MI, including atrial fibrillation, non-ST-segment elevation on ECG, chronic heart failure, and chronic obstructive pulmonary disease (COPD). Other factors such as age, anemia, atypical symptoms, syncope, hyperlipidemia, and chronic kidney disease (CKD) were also significant (as shown in Table 3).

**Table 3:** Univariate logistic regression analysis for predicting T2MI in the training set.

Variable	OR (95% CI)	P Formatted
Atrial fibrillation	2.977 (1.836-4.825)	<0.001
Non-ST-segment elevation on ECG	7.861 (5.114-12.085)	<0.001
Chronic heart failure	3.224 (1.721-6.039)	<0.001
COPD	2.646 (1.48-4.73)	0.001
Age	1.038 (1.014-1.063)	0.002
Anemia	3.171 (1.46–6.889)	0.004
No Typical symptoms	1.823 (1.207–2.753)	0.004
Syncope	2.439 (1.088–5.467)	0.030
Hyperlipidemia	2.746 (1.098–6.869)	0.031
CKD	1.788 (1.005–3.18)	0.048
Female	1.428 (0.95–2.146)	0.087
cTn	1.032 (0.929–1.145)	0.561
Current drinkers	1.19 (0.323–4.391)	0.794
Previous myocardial infarction	1.133 (0.367–3.505)	0.828
Diabetes	0.956 (0.625–1.462)	0.834
Hypertension	0.953 (0.608–1.495)	0.834
Malignant tumor	0.876 (0.187–4.108)	0.867
Current smokers	1.018 (0.476–2.176)	0.964

cTn, cardiac Troponin; COPD, Chronic obstructive pulmonary disease; CKD, Chronic Kidney Disease.

Further analysis using multivariate logistic regression revealed that atrial fibrillation (OR = 3.318, 95% CI: 1.694–6.5, *P* < 0.001), COPD (OR = 4.04, 95% CI: 1.771–9.217, *P* < 0.001), anemia (OR = 5.351, 95% CI: 1.737–16.48, *P* = 0.004), chronic heart failure (OR = 2.379, 95% CI: 1.093–5.178, *P* = 0.029), atypical symptoms (OR = 1.884, 95% CI: 1.107–3.207, *P* = 0.019), and non-ST-segment elevation on ECG (OR = 7.113, 95% CI: 4.277–11.828, *P* < 0.001) were identified as independent risk factors for T2MI. These results are summarized in Table 4. We evaluated multicollinearity among the candidate variables using the variance inflation factor (VIF). All variables demonstrated VIF values below 5, indicating the absence of severe multicollinearity within the model, as shown in Supplementary Table S1.

**Table 4:** Multivariate logistic regression analysis of risks of T2MI in training set.

Variable	Adjusted OR (95% CI)	*P* value
Chronic heart failure	2.379 (1.093–5.178)	0.029
Non-ST-segment elevation on ECG	7.113 (4.277–11.828)	<0.001
Age	1.022 (0.992–1.054)	0.154
Anemia	5.351 (1.737–16.48)	0.004
Atrial fibrillation	3.318 (1.694–6.5)	<0.001
COPD	4.04 (1.771–9.217)	<0.001
No Typical symptoms	1.884 (1.107–3.207)	0.019
CKD	1.024 (0.468–2.241)	0.952
Female	1.279 (0.758–2.158)	0.357
Malignant tumor	2.566 (0.586–11.228)	0.211
cTn	1.047 (0.936–1.172)	0.423

cTn, cardiac Troponin; COPD, Chronic obstructive pulmonary disease; CKD, Chronic Kidney.

### 3.4 Development of the prediction model and internal validation of its performance

#### 3.4.1 A nomogram model predicts high-risk T2MI populations from initial screenings

**Figure 2:**
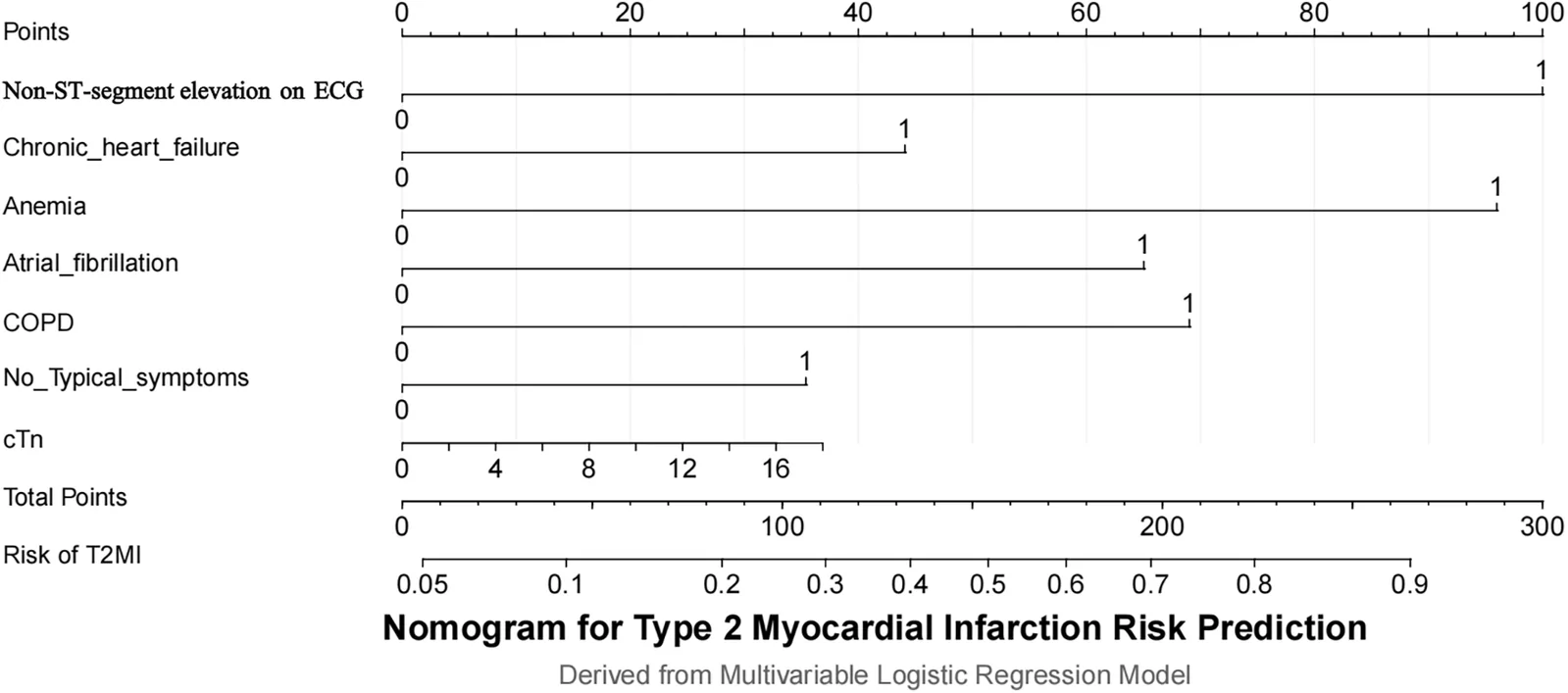
Nomogram for type 2 myocardial infarction risk predicton.

Troponin, a cornerstone biomarker for the diagnosis of myocardial infarction and the basis of this study, was retained in the final model due to its established clinical relevance, despite not achieving statistical significance in the multivariable regression analysis. In addition, we identified six other independent risk predictors: non-ST-segment elevation on ECG, chronic heart failure, anemia, atrial fibrillation, COPD, and atypical symptoms. We then constructed a nomogram based on these predictors using R. This nomogram screens the risk of T2MI in geriatric ACS patients by summing individual diagnostic factor scores into a total score, as shown in Figure 2.

#### 3.4.2 Evaluation of the predictive ability of the nomogram model

**Figure 3:**
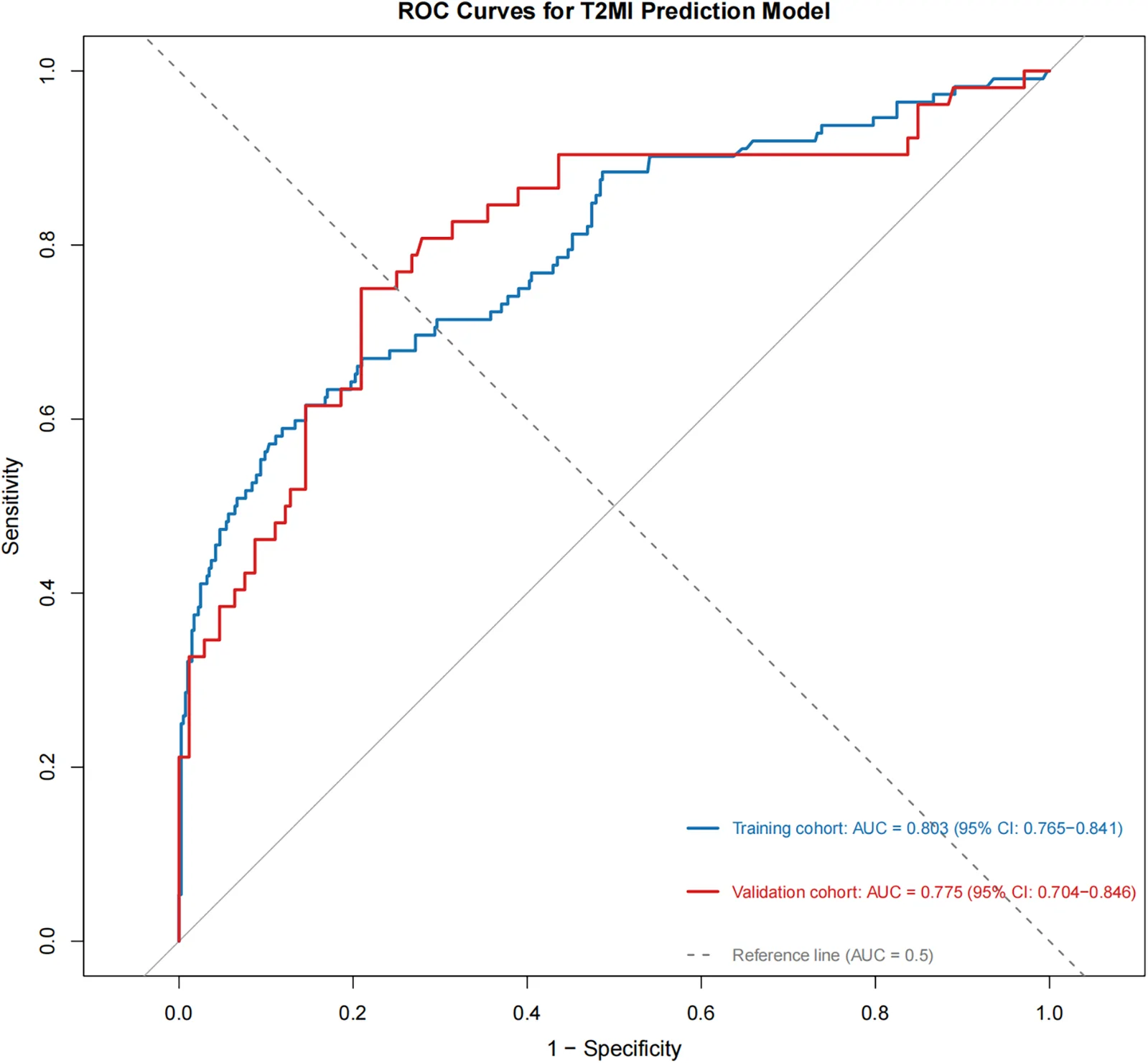
Comparison of area under the receiver operating characteristic curve (AUROC) values among different scoring systems for prediction of T2MI in the training group and validation group.

The ROC analysis showed that the nomogram had an AUC of 0.803 (95% CI: 0.765–0.841) in the training set. In the validation set, the AUC was 0.775 (95% CI: 0.704–0.846) (Figure 3). The optimal probability cutoff value was determined based on the maximum Youden index: in the training set, the optimal cutoff value was 0.287, with a sensitivity of 0.647, specificity of 0.826, positive predictive value (PPV) of 0.502, and negative predictive value (NPV) of 0.896; in the validation set, the optimal cutoff value was 0.287, with a sensitivity of 0.641, specificity of 0.783, PPV of 0.482, and NPV of 0.874 (Table 5).

**Table 5:** Discrimination and classification performance of the model in the training and validation sets.

Group	AUC (95%CI)	Sensitivity (95%CI)	Specificity (95%CI)	PPV (95%CI)	NPV (95%CI)	cut off
Training	0.803 (0.765–0.841)	0.647 (0.577–0.712)	0.826 (0.796–0.852)	0.502 (0.440–0.564)	0.896 (0.870–0.918)	0.287
Validation	0.775 (0.704–0.846)	0.641 (0.518–0.747)	0.783 (0.722–0.834)	0.482 (0.379–0.587)	0.874 (0.818–0.914)	0.287

**Figure 4:**
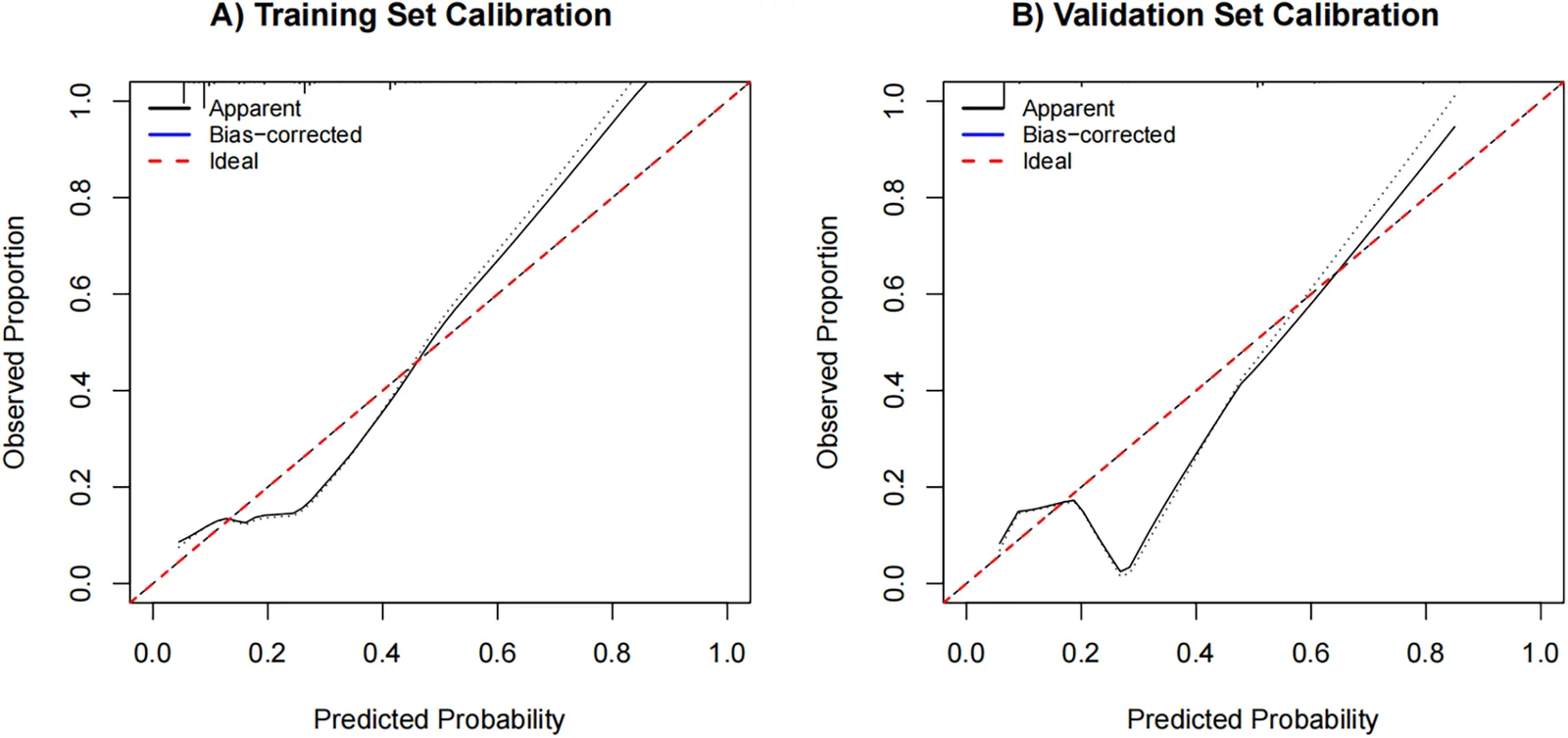
Calibration curve of the nomogram for the training group **(A)** and validation group **(B)**.

Additionally, the calibration curve for the predicted probability of T2MI in geriatric patients with ACS demonstrated concordance between the model-predicted probabilities and the observed probabilities in both the training and validation sets in Figure 4. Calibration metrics were summarized in Table 6. In the training set, the CITL intercept was 0(95%CI −0.1843, 0.1843), and the calibration slope was 1(95%CI 0.8401, 1.1599). In the independent validation set, the CITL intercept was 0.2134 (95%CI −0.1125, 0.5393), and the calibration slope was 0.8603 (95%CI 0.5995, 1.1210). The 95% confidence intervals of CITL intercept and calibration slope in the validation set included 0 and 1, respectively, demonstrating acceptable global calibration of the model. The Brier Score slightly increased from 0.1232 in the training set to 0.1405 in the validation set, suggesting mild overfitting. The calibration curve showed satisfactory agreement between predicted and observed event probabilities across most risk ranges.

**Table 6:** Calibration metrics in the training and validation sets.

Calibration metrics	Training Set	Validation Set
Brier Score	0.1232	0.1405
CITL Intercept	0 (95%CI −0.1843, 0.1843)	0.2134 (95%CI −0.1125, 0.5393)
Calibration Slope	1 (95%CI 0.8401, 1.1599)	0.8603 (95%CI 0.5995, 1.1210)

#### 3.4.3 Clinical decision evaluation of the nomogram

**Figure 5:**
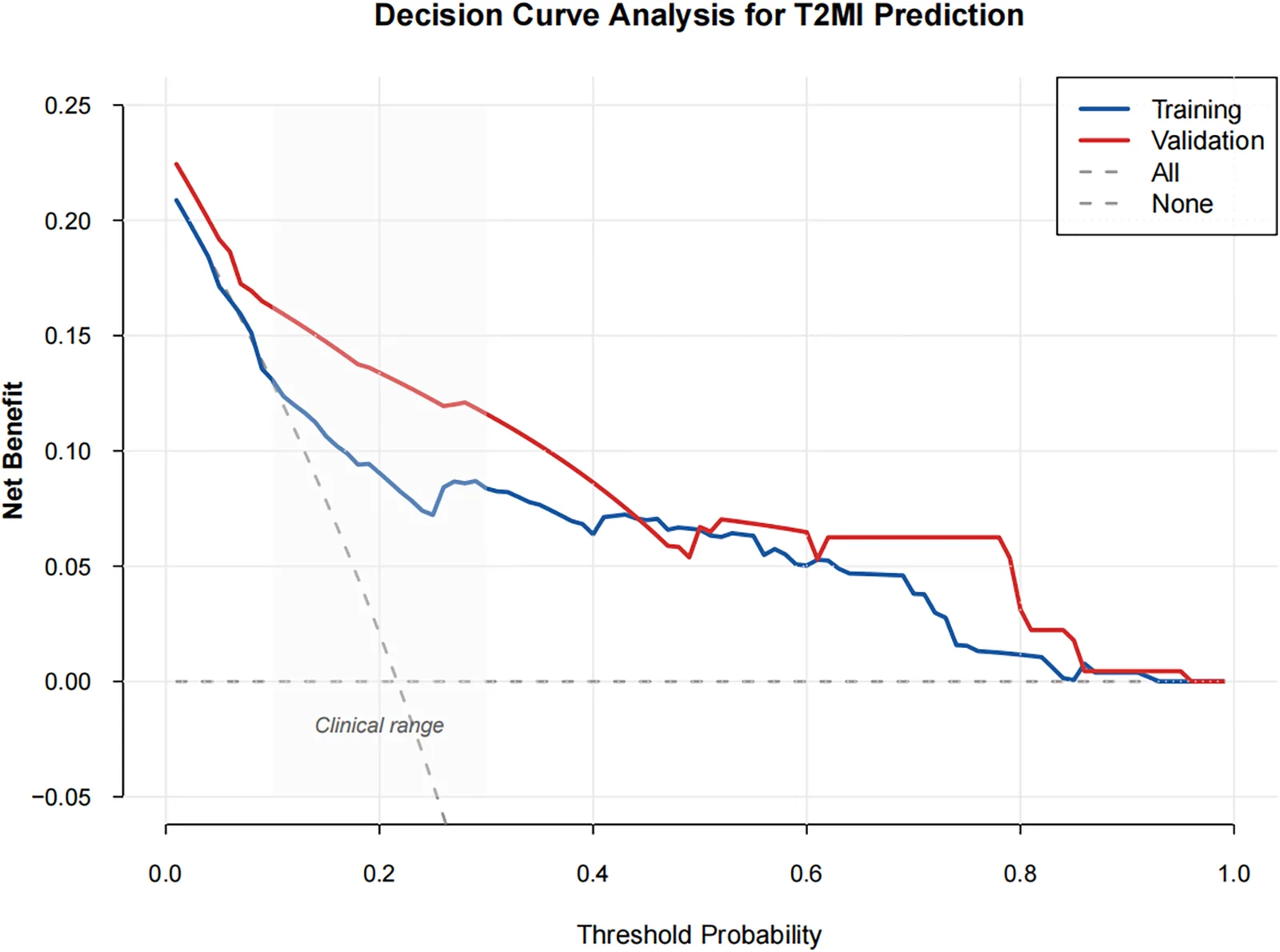
Clinical impact curves of nomogram model in the training group and validation group.

A clinical decision analysis (DCA) was conducted with the high-risk threshold probability on the horizontal axis and net benefit on the vertical axis. The high-risk threshold probability, ranging from 0 to 1, was used in the analysis. The DCA curve indicated that between threshold probabilities of 0.01 and 0.99, the nomogram model offered clinical net benefits in both training and validation datasets (Figure 5).

#### 3.4.4 Validation of model stability

After exclusion of patients with STEMI from the overall ACS cohort, ROC curve analysis demonstrated an AUC of 0.927 (95% CI: 0.899–0.955) in the training set and 0.942 (95% CI: 0.904–0.980) in the validation set, as shown in Supplementary Figure S1.

Further analysis demonstrated that the model achieved a sensitivity of 0.958, a NPV of 0.983, a specificity of 0.727, and a PPV of 0.504 in the training set; the corresponding values in the validation set were 0.971, 0.985, 0.688, and 0.540, respectively (Supplementary Table S2).

The calibration curves demonstrated close agreement between the predicted and observed probabilities (Supplementary Figures 2 and 3).

Decision curve analysis indicated that the model provided a consistently higher net benefit than default strategies across a wide range of clinically reasonable threshold probabilities, supporting its potential to offer clinical utility (Supplementary Figures S4 and S5).

## 4 Discussion

The primary objective of this study was to develop a tool to identify T2MI among geriatric patients with ACS, with the aim of providing additional support for clinical decision-making in the emergency department setting. Given the differing therapeutic pathways for T1MI and T2MI, the early identification of T2MI is of considerable significance in guiding its clinical management. This study found that T2MI accounted for over one fifth of ACS patients who presented at the chest pain center of the emergency department. Previous studies reported that T1MI and T2MI together account for about 98% of AMI cases (32). T2MI is one classification of AMI and accounts for approximately 20%–30% of cases. It is more prominent in the geriatrics and in patients with multiple comorbidities (3, 4). Notably, due to immunosenescence (33), multiple systemic comorbidities (34), and atypical symptoms in the geriatrics, their clinical manifestations are more complex, which further increases the difficulty of rapid classification for geriatric ACS patients. Therefore, by utilizing clinical information rapidly obtainable in the emergency department and incorporating the characteristics of the emergency chest pain center, the present study developed and validated a nomogram model that can assist in the early screening for T2MI in elderly patients with ACS.

In addition, T1MI focuses on vascular recanalization, whereas T2MI focuses on the treatment of the primary disease (6, 31). Clear guideline recommendations exist for treating T1MI and angina pectoris; however, evidence-based guidance for T2MI treatment is still insufficient (31, 35). This underscores the critical importance of accurately diagnosing T2MI in geriatric ACS patients. Precise diagnosis supports better treatment strategies and reduces the risk of invasive procedures and related comorbidities. Therefore, considering the characteristics of emergency department and clinical features of geriatric patients, this study developed a nomogram model to help identify T2MI in geriatric ACS patients. The model included six independent risk factors (non-ST-segment elevation on ECG, chronic heart failure, anemia, atrial fibrillation, COPD, and atypical symptoms) plus cTn, an essential biomarker for the diagnosis of myocardial infarction. These factors, which demonstrated good discrimination, calibration, and clinical utility, performed well in the training and validation sets.

T2MI is defined as myocardial ischemia resulting from an imbalance between myocardial oxygen supply and demand. It is frequently precipitated by conditions such as anemia, tachyarrhythmia, or hypotension or shock, and respiratory failure (23). T2MI is more frequently characterized by non-ST-segment elevation on ECG, however, this electrocardiographic finding lacks specificity. While non-ST-segment elevation can also be present in NSTEMI resulting from T1MI and is not pathognomonic for T2MI, relying solely on non-ST-segment elevation on ECG cannot differentiate T1MI from T2MI，however, early identification and differential diagnosis may be facilitated by integrating other clinical information. cTn is commonly used for the differential diagnosis of myocardial infarction and myocardial injury (5). However, in clinical practice, the distributions of troponin concentrations for T1MI and T2MI significantly overlap (31, 35). In the emergency setting, therefore, high-sensitivity cTn can only indicate myocardial injury but cannot distinguish between types of myocardial infarction (36, 37). Therefore, the differentiation between T1MI and T2MI requires a comprehensive consideration incorporating other clinical information. Accordingly, the model developed in this study offers an additional approach for differential diagnosis.

The following variables may be interpreted from oxygen supply-demand imbalance and the decline in physiological reserve in elderly individuals. This study found that atrial fibrillation is an important factor for identifying T2MI in geriatric patients with ACS. Rapid AF may increase myocardial oxygen consumption and shorten diastolic coronary perfusion time, thereby exacerbating myocardial oxygen supply-demand imbalance (7, 38–40). In addition, chronic heart failure is one of the risk factors for T2MI, as it may reduce myocardial reserve, rendering elderly individuals more susceptible to oxygen supply-demand imbalance and T2MI under hemodynamic stress (35, 37, 41–44). These findings support Curcio et al.'s (3) concept that T2MI should be considered a syndrome predominantly affecting the geriatrics. Furthermore, this study found that COPD, a common chronic comorbidity, increases the risk of T2MI in geriatric patients with ACS. COPD may, through hypoxemia and sympathetic activation, reduce myocardial oxygen supply and increase myocardial oxygen consumption, thereby precipitating T2MI (8, 45–47). Anemia is common in geriatric patients (42). It causes insufficient hemoglobin and directly reduces the blood's oxygen-carrying capacity, which results in decreased myocardial oxygen supply. Anemia also increases the cardiac load to compensate for the insufficient oxygen supply, thereby exacerbating the imbalance between myocardial oxygen supply and demand (48, 49).

Moreover, studies show that typical chest pain occurs infrequently in T2MI patients (31). Instead, symptoms mainly manifest atypically as shortness of breath, fatigue, and discomfort in the upper abdomen (35, 50, 51). In elderly patients, reduced pain sensitivity, age-related functional frailty, and multimorbidity may further contribute to atypical symptoms, thereby masking the true manifestations of ischemia (34, 52–54). This suggests that Emergency physicians should be vigilant for atypical chest pain in geriatric patients, especially those suspected of ACS, and conduct further screening for T2MI.

Previous studies have demonstrated that, The Extended Neumann Score (16). comprising sex, radiating chest pain, initial hs-cTnI level, and heart rate >120 beats/min, serves as an auxiliary tool for subtype classification in patients with acute myocardial infarction and is mainly used to distinguish T1MI from T2MI. However, its discriminatory capacity is limited, and it was not developed specifically for elderly patients with ACS (16). The T2-risk score is applied to patients with an established diagnosis of T2MI and primarily predicts 1-year mortality or recurrent myocardial infarction, rather than serving for the early identification of T2MI in the emergency department. This score comprises nine variables: age, ischemic heart disease, heart failure, diabetes mellitus, electrocardiographic myocardial ischemia, heart rate, anemia, estimated glomerular filtration rate (eGFR), and the maximum cardiac troponin concentration. Therefore, it is more suitable for prognostic stratification following diagnostic confirmation. As the maximum cardiac troponin concentration is not always obtainable at the initial emergency department assessment, the time point of its application differs from that of tools designed for early T2MI screening based on information acquired at the index presentation (17). The HEART and EDACS scores are primarily utilized to identify chest pain patients at low short-term risk of major adverse cardiovascular events (MACE) who may be suitable for early discharge, whereas the GRACE score is primarily employed for the assessment of mortality and ischemic event risk in patients with confirmed or suspected ACS. The outcomes of these tools are not etiological classifications of T2MI, consequently, they cannot be directly applied to differentiate T2MI, T1MI, and non-ischemic myocardial injury (11, 13, 14). Furthermore, age carries a substantial weight in certain chest pain scores, and elderly patients are frequently difficult to classify as low-risk due to age, comorbidities, and abnormal baseline cTn levels. An age-subgroup analysis of the HEART Pathway demonstrated that the proportion of patients identified as low-risk was lower among elderly patients, and that the pathway did not significantly reduce hospitalizations in this age group, suggesting that the triage efficiency of generic chest pain tools may be limited in the elderly emergency population (11). CoDE-ACS is applied to emergency patients with suspected myocardial infarction, integrating high-sensitivity cardiac troponin (hs-cTn) with clinical information—including age, sex, time from symptom onset, chest pain, prior ischemic heart disease, dyslipidemia, heart rate, systolic blood pressure, Killip class, electrocardiographic ischemia, renal function, and hemoglobin—through machine learning to estimate the individualized probability of myocardial infarction, its number of variables and computational burden exceed those of simple clinical scores, and the primary objective of the model is not the direct differentiation between T1MI and T2MI. Furthermore, its development and validation are dependent on a specific hs-cTnI assay platform (18). Recent machine learning-based myocardial injury classification models have further expanded the classification scope, targeting patients with suspected ACS. By utilizing multidimensional data primarily derived from electronic health records, these models first distinguish acute myocardial injury patterns from no myocardial injury or chronic myocardial injury patterns, and subsequently classify acute myocardial injury into acute non-ischemic myocardial injury, T1MI, and T2MI. Additionally, an independent model was established to predict 30-day mortality or myocardial infarction (MI). This approach utilized algorithms such as XGBoost and deep learning, the authors noted that further external validation is still required to evaluate its clinical applicability. Compared with simplified scores that employ only a few conventional variables, its data and computational requirements are higher (19). MI^3^, employing a different simplified machine learning strategy, primarily integrates age, sex, serial hs-cTnI concentrations, and the time interval between two blood samplings to estimate the individualized probability of a diagnosis of T1MI in patients with suspected MI. Although it requires relatively few input variables, at least two cardiac troponin measurements are needed, furthermore, the model was not originally developed for the direct differentiation between T1MI and T2MI, and is therefore inconsistent with the objective of T2MI screening in the present study (20). Additionally, studies have combined biomarker approaches to enhance the discriminatory capacity for myocardial infarction subtypes. Neumann et al. evaluated 29 biomarkers in patients with suspected MI and ultimately selected Apo A-II, NT-proBNP, copeptin, and hs-cTnI for the differentiation between T1MI and T2MI. Although this method demonstrates a certain discriminatory ability, its direct application in rapid emergency department screening is limited by high laboratory requirements, the small number of T2MI cases in the study, and the fact that only internal validation was performed (21).

In contrast to the aforementioned tools, the present study focuses on elderly patients with ACS, a special population characterized by multimorbidity, complex clinical presentations, and substantial heterogeneity (55, 56). The objective was to construct a nomogram, based on rapidly obtainable clinical variables, to facilitate rapid screening for T2MI at the initial emergency department presentation. This tool, built from straightforward variables accessible in emergency settings, allows for more precise individualized risk assessment, potentially improving risk stratification and clinical management in this complex population. It must be emphasized that direct numerical comparison of the area under the receiver operating characteristic curve (AUC) values of these models is inappropriate, given the differences in their target populations, prediction tasks, outcome definitions, prediction time points, timing of variable ascertainment, and validation designs. Therefore, the practical value of a model should be comprehensively evaluated by integrating its specific clinical purpose, the accessibility of incorporated variables, and the applicable settings, rather than being judged solely by the magnitude of its area under the AUC value.

This study has several limitations. Firstly, it is a retrospective study and cannot establish causal relationships; Secondly, the data in this study were collected from a single regional medical center in Shanghai, and the sample size was limited. This may limit the generalizability of the results. Therefore, researchers need to conduct multi-center studies to further validate and improve the model's clinical utility. Thirdly, we conducted internal validations of the nomogram using ROC curves, calibration plots, and decision curve analysis, which demonstrated relatively accurate predictive performance in diagnosing T2MI among geriatric patients. However, further prospective studies are necessary for external validation. Fourth, this study did not account for patients' prior medication use. Such medications could confound clinicians' assessment of presenting symptoms; for instance, analgesics may elevate the pain threshold, potentially leading to the misclassification of atypical chest pain. Consequently, residual confounding cannot be excluded. Fifth, the primary objective of this study was to enable early risk assessment for T2MI, not to rule out T1MI. Its clinical utility lies in alerting clinicians to the possibility of T2MI, thereby prompting enhanced differential diagnosis and the early initiation of interventions aimed at correcting myocardial oxygen supply–demand imbalance. Future integration of dynamic electrocardiographic monitoring, imaging data, and additional clinical biomarkers could further refine the model's capacity to support clinical decision-making. Sixth, this study included only patients who underwent coronary angiography and did not include patients who, in routine clinical practice, were still at the stage of determining whether invasive evaluation should be performed. Therefore, the present study cannot establish whether the model can reduce unnecessary coronary angiography. Seventh, this study did not compare the AUC, calibration, or clinical net benefit between models with and without cardiac troponin. Therefore, it remains unclear whether retaining cardiac troponin ultimately improves the predictive performance of the model.

The clinical implementation of this model is feasible within routine emergency department workflows, as it can be applied following initial history-taking and basic laboratory testing. Integration into the Electronic Medical Record (EMR) system or a dedicated chest pain evaluation platform could enable automated risk calculation, thereby facilitating real-time risk stratification and supporting clinical decision-making. Future research could leverage machine learning techniques, utilizing the key variables identified here, to achieve high-dimensional data integration and enhance the model's predictive accuracy, robustness, and clinical utility.

## 5 Conclusion

This study used routine clinical data to construct and validate a nomogram for the rapidly help to screen T2MI in geriatric patients with ACS, which may help clinicians promptly make personalized decisions based on each patient's risk profile.

## Data Availability

The original contributions presented in the study are included in the article/Supplementary Material, further inquiries can be directed to the corresponding author/s.

## Supplementary material

SUPPLEMENTARY FIGURE S1 Comparison of area under the receiver operating characteristic curve (AUROC) values among different scoring systems for prediction of T2MI in the training and validation group of validation of model stability.

SUPPLEMENTARY FIGURE S2 Calibration curve of the nomogram for the training group of validation of model stability.

SUPPLEMENTARY FIGURE S3 Calibration curve of the nomogram for the validation group of validation of model stability.

SUPPLEMENTARY FIGURE S4 Clinical impact curves of nomogram model in the training group of validation of model stability.

SUPPLEMENTARY FIGURE S5 Clinical impact curves of nomogram model in the validation group of validation of model stability.
