# Supplementary material for: An easy-to-use model for screening type 2 myocardial infarction in geriatric patients with acute coronary syndrome: a retrospective cohort study
Source: Front Med (Lausanne). 2026 Aug 27;13:1907612. doi: 10.3389/fmed.2026.1907612 (PMC13557950; doi:10.3389/fmed.2026.1907612)
Supplement: Supplementary file 1 [file Table1.docx]

**Supplementary Table 1. Multicollinearity assessment using variance inflation factors**

| **Variable** | **Variance Inflation Factor (VIF)** | **Collinearity Status** |
| --- | --- | --- |
| Non-ST-segment elevation on ECG | 1.12 | Acceptable |
| Chronic_heart_failure | 1.05 | Acceptable |
| age | 1.11 | Acceptable |
| Anemia | 1.14 | Acceptable |
| Atrial_fibrillation | 1.09 | Acceptable |
| COPD | 1.12 | Acceptable |
| No_Typical_symptoms | 1.11 | Acceptable |
| CKD | 1.11 | Acceptable |
| Female | 1.11 | Acceptable |
| malignant_tumor | 1.03 | Acceptable |
| cTn | 1.06 | Acceptable |
| VIF > 10 indicates severe multicollinearity | | |
| VIF > 5 indicates moderate multicollinearity | | |
| VIF ≤ 5 indicates acceptable multicollinearity | | |
