# Supplementary material for: An easy-to-use model for screening type 2 myocardial infarction in geriatric patients with acute coronary syndrome: a retrospective cohort study
Source: Front Med (Lausanne). 2026 Aug 27;13:1907612. doi: 10.3389/fmed.2026.1907612 (PMC13557950; doi:10.3389/fmed.2026.1907612)
Supplement: Supplementary file 2 [file Table2.docx]

### Table Supplementary Table 2 confusion matrix: training and validation

| Data | AUC (95%CI) | Accuracy (95%CI) | Sensitivity (95%CI) | Specificity (95%CI) | PPV (95%CI) | NPV (95%CI) | cut off |
| --- | --- | --- | --- | --- | --- | --- | --- |
|  |  |  |  |  |  |  |  |
| Training | 0.927 (0.899-0.955) | 0.778 (0.729-0.823) | 0.958 (0.911 - 1.000) | 0.727 (0.671 - 0.782) | 0.504 (0.419 - 0.588) | 0.983 (0.965 - 1.000) | 0.224 |
| Validation | 0.942 (0.904-0.980) | 0.766 (0.683-0.836) | 0.971 (0.916 - 1.000) | 0.688 (0.594 - 0.782) | 0.540 (0.417 - 0.663) | 0.985 (0.955 - 1.000) | 0.224 |
